# With great power comes great responsibility: the UK Biobank sleep dataset

**DOI:** 10.1093/sleep/zsag096

**Published:** 2026-04-06

**Authors:** Tamar Sofer, Daniel J Gottlieb

**Affiliations:** Department of Medicine, Harvard Medical School, Boston, MA, United States; Division of Sleep and Circadian Disorders, Brigham and Women’s Hospital, Boston, MA, United States; Cardiovascular Division, Beth Israel Deaconess Medical Center, Boston, MA, United States; Department of Biostatistics, Harvard T.H. Chan School of Public Health, Boston, MA, United States; Department of Medicine, Harvard Medical School, Boston, MA, United States; Division of Sleep and Circadian Disorders, Brigham and Women’s Hospital, Boston, MA, United States

The UK Biobank (UKB) is a study following ~500 000 British individuals, mostly White/of European ancestry [1]. It has already transformed health research with the availability of genetic data on virtually all participants, as well as other omics, large scale imaging, and detailed health measures [2–4]. In this issue of *SLEEP*, Tse et al. [5] report the creation of a comprehensive questionnaire-based dataset for sleep research in UKB, available for about ~185 000 of UKB participants. This resource is poised to accelerate observational studies of sleep, given the size of UKB and the ease of gaining access to UKB data.

Sleep research studies enabled by the UKB cohort data include descriptive and association analyses linking sleep behaviors and disorders with other health outcomes, environmental and lifestyle exposures, and extensive genetic data, including whole genome sequencing. These data have permitted genetic analyses of sleep traits including single variant and variant set association analyses, estimation of heritability (overall genetic contribution to a phenotype), genetic correlations, and Mendelian randomization analyses. While these types of analyses were already possible using existing UKB data [6–9], they utilized a limited set of “simple” sleep traits, typically measured via a single question per domain. While data from linked electronic health records (EHR) are also available, it is often insufficient for appropriately studying sleep. Consider, for example, obstructive sleep apnea (OSA) and insomnia, two highly prevalent sleep disorders. While OSA prevalence estimates vary widely by diagnostic criteria and by country, it has an estimated adult population prevalence of at least 6%–17% in west European countries [10], with many estimates considerably higher [11], and a large sex bias [11]. OSA is also known to be underdiagnosed, with disparities by groups of different characteristics, including race, insurance status, education, and others [12]. Previous UKB papers using data from EHR or self-reported doctor diagnosed sleep apnea [13, 14] reported an OSA prevalence of about 1% among females and 2.8% among males. These numbers are much lower than the expected OSA prevalences, likely reflecting the known OSA underdiagnosis. This led some researchers to use snoring, an OSA symptom, as a proxy for OSA in order to increase power for detecting genetic associations with OSA [15]. The new sleep questionnaire enhancement data is modeled on a validated questionnaire instrument, the Berlin questionnaire [16], to assess OSA, although the reported approach to scoring differs somewhat from the original. The results suggest 7.9% of females and 8% of males have high risk of OSA.

Prior to the introduction of the new sleep questionnaire enhancement data, insomnia was usually assessed by the question “Do you have trouble falling asleep at night or do you wake up in the middle of the night?” with the response “usually” used to define a case [17]. This resulted in prevalence of 32% in females and 24% in males. In the new questionnaire, chronic insomnia disorder was defined using the Sleep Condition Indicator [18], resulting in a prevalence of 14.4% (17.1% in females and 10.7% in males). The prevalence estimates from the new questionnaire are closer to the 12.4% sex-combined prevalence reported in a recent meta-analysis, citing DSM diagnosis by a clinician as the gold standard [19].

The new sleep questionnaire data provide many opportunities for sleep research including for less common phenotypes such as sleep–wake phase disorders and sleepwalking. Research directions include analyses of data that typically requires large datasets such as genetics and omics data. Importantly, genome-wide association studies (GWASs) using “crude” phenotypes have been very successful in identifying genomic loci associated with traits, often to a larger extent than small genetic studies with well-phenotyped traits. For example, GWASs of OSA with hundreds of thousands of individuals, using EHR-based definitions of OSA, resulted in more replicated genetic associations than studies of quantitative measures of OSA from tens of thousands of individuals [20–23].

While the expanded and detailed sleep UKB dataset provides many opportunities, it also poses some challenges for the sleep research community and beyond. From a statistical standpoint, there is a selection bias whereby UKB sleep questionnaire participants are not a random sample of the UKB cohort. This compounds the known bias in UKB participation, where the set of all study participants does not represent the UK population, leading to biased effect estimates, e.g., in genetic association analyses [24]. Table 1 provides an overview of a few potential biases that must be considered in analyses of UKB data. Misclassification bias, which may be evidenced by apparent prevalences that are inconsistent with known population prevalence of the underlying target condition, can be due to selective recall, or various limitations in how individuals interpret the questionnaire items [25]. In the context of a population-based study, selection bias occurs when the available population for a study does not represent the relevant general population [26]. Both types of biases can lead to poor estimation, with direction of bias depending on specific conditions [27]. While such biases may be relevant in many other datasets, a large dataset such as the UKB will provide great power and is expected to generate effect sizes with smaller standard errors and more “exciting” *p*-values, therefore having higher potential to generate widely disseminated but misleading results. It is therefore incumbent on investigators to carefully consider potential sources of bias in their interpretation of results.

**Table 1:** Potential biases impacting sleep research studies using UKB data

Name of bias	Meaning and implications
Misclassification bias	A condition such as OSA or insomnia may be misclassified. If misclassification is not systematic, association results may be shrunk towards the null. If systematic (e.g. individuals with OSA are more likely to be misclassified compared to those without OSA), the bias can be driven to either direction, depending on the details. With questionnaire data, systematic misclassification bias can be due to selective recall, among other reasons.
Selection bias	Selection bias occurs when a given study population is not representative of the target population. In case–control study it could be due to inappropriate selection of controls into the study. In cohort studies selection bias may occur when the cohort is not a random sample of the target population. For example, individuals who feel especially tired during the day may be more likely to respond to a sleep questionnaire, leading to inflated estimates of population prevalence of excessive daytime sleepiness (EDS) and of OSA, a cause of EDS.
Collider bias	A type of selection bias where the study population is selected (either by design or by participation choice) based on a condition, or a variable correlated with a condition, that is caused by both the exposure and outcome of interest in an analysis. For example, individuals who are focused on living healthier lifestyle and individuals who have poor sleep may be more likely to respond to a sleep questionnaire, and this can induce collider bias in association analysis of lifestyle measures and poor sleep phenotypes.

Here are three challenges that the sleep research community may face with the introduction of this new dataset, and recommendations for professional sleep societies and journals to address these:

Challenge 1: Some of the analyses using UKB data may not properly assess and account for potential biases in the dataset, resulting in incorrect association effect estimates, potentially leading to flashy yet misleading titles.

Recommendation 1: Researchers should prioritize development of benchmarking, best practices, and other methodological work to develop guidelines for analysis of UKB data. Sleep journals may consider a special topic issue to encourage, promote, and advertise such research.

Challenge 2: The number of manuscripts based on UKB data that will be submitted for publications in sleep journals will increase, and likely increase exponentially. This will burden the editorial and peer review systems.

Recommendation 2: Sleep-focused journals may prepare for this increase in submissions by potentially increasing and restructuring editorial teams to have appropriate dedicated staff familiar with the UKB datasets and best analytical practices, including for analysis of genetic and other omics data.

Challenge 3: The number of scientists rushing into the field of sleep research will increase. Some of these scientists, as well as peer reviewers of their work, may not have good understanding of sleep, or, conversely, of genetic and omics data analyses which are easily conducted using UKB data.

Recommendation 3: Societies should welcome new researchers in the field and prioritize developing and executing workshops on sleep epidemiology in general and on special topics, including workshops focused on analyzing sleep data in the UKB.

The addition of new sleep data to UKB is poised to increase the volume of and interest in sleep research, from both established sleep researchers and the broader research community. Ensuring that these changes happen while standards of rigor are maintained may be challenging, but despite these anticipated growing pains, the field of sleep epidemiology and sleep research will likely expand in scope, methodologies, and rigor.

## Disclosure statement

*Financial disclosure*: DJG reports receiving fees from Lilly USA, Inc., and Takeda Development Center Americas, Inc. for consulting; receiving fees from Apimend, Inc. for serving in a Data Monitoring Committee, and receiving fees from Signifier Medical Technologies for serving in a Medical Advisory Board.

*Non-financial disclosure*: None declared.
